# Direct 16S/18S rRNA Gene PCR Followed by Sanger Sequencing as a Clinical Diagnostic Tool for Detection of Bacterial and Fungal Infections: a Systematic Review and Meta-Analysis

**DOI:** 10.1128/jcm.00338-23

**Published:** 2023-06-27

**Authors:** Pavel Drevinek, Regina Hollweck, Michael G. Lorenz, Michael Lustig, Thomas Bjarnsholt

**Affiliations:** a Department of Medical Microbiology, 2nd Faculty of Medicine, Charles University and Motol University Hospital, Prague, Czech Republic; b Novustat GmbH, Wollerau, Switzerland; c Molzym GmbH & Co. KG, Bremen, Germany; d Department of Clinical Microbiology, Centre for Diagnostics, Rigshospitalet, Copenhagen, Denmark; e Costerton Biofilm Center, University of Copenhagen, Copenhagen, Denmark; Vanderbilt University Medical Center

**Keywords:** agnostic molecular diagnosis, added value of sequencing, bacterial meningitis, change of antibiotic treatment, culture-negative infections, fastidious and rare pathogens, infectious endocarditis, joint infections, nongrowing pathogens, sepsis

## Abstract

rRNA gene Sanger sequencing is being used for the identification of cultured pathogens. A new diagnostic approach is sequencing of uncultured samples by using the commercial DNA extraction and sequencing platform SepsiTest (ST). The goal was to analyze the clinical performance of ST with a focus on nongrowing pathogens and the impact on antibiotic therapy. A literature search used PubMed/Medline, Cochrane, Science Direct, and Google Scholar. Eligibility followed PRISMA-P criteria. Quality and risk of bias were assessed drawing on QUADAS-2 (quality assessment of diagnostic accuracy studies, revised) criteria. Meta-analyses were performed regarding accuracy metrics compared to standard references and the added value of ST in terms of extra found pathogens. We identified 25 studies on sepsis, infectious endocarditis, bacterial meningitis, joint infections, pyomyositis, and various diseases from routine diagnosis. Patients with suspected infections of purportedly sterile body sites originated from various hospital wards. The overall sensitivity (79%; 95% confidence interval [CI], 73 to 84%) and specificity (83%; 95% CI, 72 to 90%) were accompanied by large effect sizes. ST-related positivity was 32% (95% CI, 30 to 34%), which was significantly higher than the culture positivity (20%; 95% CI, 18 to 22%). The overall added value of ST was 14% (95% CI, 10 to 20%) for all samples. With 130 relevant taxa, ST uncovered high microbial richness. Four studies demonstrated changes of antibiotic treatment at 12% (95% CI, 9 to 15%) of all patients upon availability of ST results. ST appears to be an approach for the diagnosis of nongrowing pathogens. The potential clinical role of this agnostic molecular diagnostic tool is discussed regarding changes of antibiotic treatment in cases where culture stays negative.

## INTRODUCTION

Molecular analysis of microbial pathogens by antigens, antibodies, or nucleic acids is an important rapid complement to culture diagnosis (1–4). Rapid analysis represents a major advantage over culture, which generally takes 1 to 2 days or, in the case of fastidious microorganisms, up to several weeks (5). A timely initiation of antibiotic treatment is critical for the prospective outcome of ill patients. Also, a change from broad- to narrow-spectrum antibiotics reduces antibiotic resistance. Thus, molecular methods constitute an important aid in decision-making for treatment of critically ill patients (6).

There are two principal approaches of nucleic acid-based methods. Syndromic panel systems target a limited number of pathogens characteristic of a particular disease (7) and deliver results usually within a few hours. A major limitation of this approach constitutes the aspect that pathogens beyond the panel are missed. Agnostic approaches use sequencing of a single conserved gene, several genes, or the whole genomes of microorganisms (8, 9). Sequences are compared with libraries of known genes, which provides the identity of pathogens and, in the case of metagenomics, other relevant determinants, such as antibiotic resistance. Agnostic sequencing can identify any pathogen, including common, new, rare, fastidious, or nongrowing microorganisms (9). Major drawbacks include the detection of irrelevant contaminants, limited use of primarily nonsterile samples, intensive hands-on demand, and an extended turnaround time.

In the present study, for the first time, the current literature on the only European certificate of conformity (CE)-marked, culture-independent rRNA gene Sanger sequencing system, whose brand name is SepsiTest (ST; Molzym, Germany), is summarized. ST was approved in 2008 and, until now, remains the only Sanger sequencing-based direct diagnostic system.

ST comprises the isolation of microbial DNA from fluid and tissue specimens, followed by PCR or real-time PCR amplification of the hypervariable V3-V4 and V8-V9 regions of the 16S and 18S rRNA genes of bacteria and fungi, respectively. After approximately 4 h, PCR results are available. If positive, two seminested amplicon sequencing reactions are performed for Gram-positive and Gram-negative bacteria, as well as another reaction for fungi. A BLAST analysis of the sequences is used to identify the taxa (10–12). For this, NCBI BLAST or the company’s own search tool and library of edited sequences from cultured species, SepsiTest-BLAST, can be used (www.sepsitest-blast.com). The ST workflow is implemented with the SepsiTest-UMD, UMD-SelectNA, and Micro-Dx platforms, which differ regarding manual, semiautomated, and fully automated DNA extraction, respectively. Amplification and sequencing are identical among the three platforms. The principle of extraction includes removal of free-floating DNA. Chaotropic conditions lyse human cells and release their DNA, which together with DNA from dead microorganisms, is degraded by an added DNase. Finally, microorganisms are collected by centrifugation (SepsiTest-UMD and UMD-SelectNA) or filtration (Micro-Dx) and lysed by a reagent, followed by purification of microbial DNA. Consequently, positive PCR results indicate the presence of living microorganisms (13). According to the manufacturer, the analytical sensitivities for selected Gram-positive and Gram-negative bacteria and Candida species vary between 20 and 80 CFU/mL. At this time, sequencing analysis of various clinical and other specimens has identified more than 1,300 bacteria and fungi (12).

This review summarizes the current literature on ST, determines its accuracy and the added value of the detection of nongrowing pathogens by meta-analysis, and discusses the clinical impact of the results.

## LITERATURE SELECTION, STUDY QUALITY ASSESSMENT AND DATA ANALYSIS

### Literature search.

A literature search was conducted according to the PRISMA-P criteria of systematic reviews (14) using PubMed/Medline, Cochrane, ScienceDirect, and Google Scholar. References to studies employing ST were extracted from reviews and original papers on sepsis published from 2018 to 2022 to account for recent and prior multiplex test systems with references to ST. Search terms comprised the strings “culture-independent” [OR] “direct” [AND] “multiplex” [AND] “PCR based assay” [OR] “PCR” [AND] “sepsis.” Another search run spanning from 2008 until December 2022 utilized the brand names of the system (SepsiTest, SelectNA, and Micro-Dx) and the company name to account for citations regarding other diseases.

Citations were screened by title and abstract by an author and were included for full text analysis if they comprised (i) full articles in peer-reviewed journals (including preprints), (ii) accessible data of reviews allowing statistical analyses of accuracy, (iii) a culture-independent diagnostic approach, and (iv) infections of sterile body parts. Exclusion criteria for eligible studies entailed (i) studies on a particular species or higher taxonomic subgroup of microorganisms (e.g., Gram-positive bacteria), (ii) studies not following the intended purpose of ST according to the CE-IVD (*in vitro* diagnostic) declarations (i.e., studies were excluded if they met one or more of the following criteria: use of frozen samples, samples beyond recommended quantities, or samples left over from previous microbiological or chemical analyses), (iii) populations without microbiological characterization of the specimens used, and (iv) populations without suspected bacterial or fungal infections. The exclusion of citations and selection of eligible studies from the search was controlled by another author. Discussion among all authors led to an agreement on eligible studies for meta-analysis.

### Data extraction.

Study characteristics included (i) country, (ii) disease, (iii) specimen, (iv) type, (v) aims, and (vi) clinical diagnosis. Methodologies encompassed (i) type of analysis (16S or 18S rRNA gene), (ii) ST platform, (iii) PCR assay, (iv) reference standard, (v) additional tests, (vi) evaluation of ST-positive, culture-negative results of clinical relevance according to the judgement of the papers’ authors (for the purpose of this review, the results were classified as being of “added value” according to Tkadlec et al. [15]), and (vii) change of antibiotic treatment on the basis of ST results. Data were extracted and agreed upon by two authors independently.

### Risk of bias and study quality assessment.

Bias and applicability were evaluated according to QUADAS-2 (quality assessment of diagnostic accuracy studies, revised) criteria (16). Accordingly, the risk of bias, referring to limitations or shortcomings in the design or conduct of studies, was evaluated for four domains: participant selection, index test, reference standards, and flow and timing between index test and standards. The assessment of applicability concerns regarding the match of the review objectives was conducted for the first three domains. The data of each study were weighted using the MetaXL add-on for Microsoft Excel (17). Evaluations were performed independently by two authors, as well as being reviewed, discussed, and adjusted in accordance with the two reviewers. If needed, a third, external expert was integrated to resolve disagreements. The proportions of false positives and false negatives per total results were determined for ST and the reference standard. Technical failure of ST was calculated in proportion to all samples.

### Data preparation and statistical analysis.

Meta-analyses across the eligible studies were conducted assessing the sensitivity, specificity, diagnostic odds ratio (DOR), and added value of ST. Continuity correction was applied in case of zero event frequencies. Statistical heterogeneity as a measure of clinical diversity effects among studies, variability in study design, and risk of bias rather than being effects by chance was evaluated using Cochran’s *Q* test and the quantitative *I*^2^ statistic of study heterogeneity in a random-effects model applying inverse variance weighting of the studies. *I*^2^ estimates the percentage of variation among all studies due to heterogeneity rather than chance; an *I*^2^ value of >50% was considered to show substantial heterogeneity.

Sensitivity is defined as congruently positive ST and positive culture results per total positive culture results. As far as provided in the papers, clinically meaningful positive results were used, whereas false-positive culture and ST results were regarded as contaminants and counted as negative results. Analogously, specificity was the proportion of congruently negative ST plus negative culture per total negative culture results. With infectious endocarditis, ST results were set into proportion with the numbers of Duke-classified definitive (sensitivity) and nondefinitive (specificity) infectious endocarditis patients as above. Confidence intervals (95% CI) were calculated using the exact Pearson-Clopper method (18).

DOR is a measure of test data accuracy that combines sensitivity and specificity into a single value, ranging from 0 to infinity, with higher values indicating a better discriminatory test performance (higher accuracy). A receiver operating characteristic (ROC) curve was performed for bivariate analysis of sensitivity and specificity. ROC curves summarize the overall test performance of sensitivity and specificity and evaluate the strength of heterogeneity (19). Meta-analyses were conducted using the tidyverse, meta, metafor, and dmetar packages of the R 3.6.3 software (20).

## APPLICATIONS AND PERFORMANCE OF SEPSITEST PLATFORM IN CLINICAL STUDIES

### Study search.

**FIG 1:**
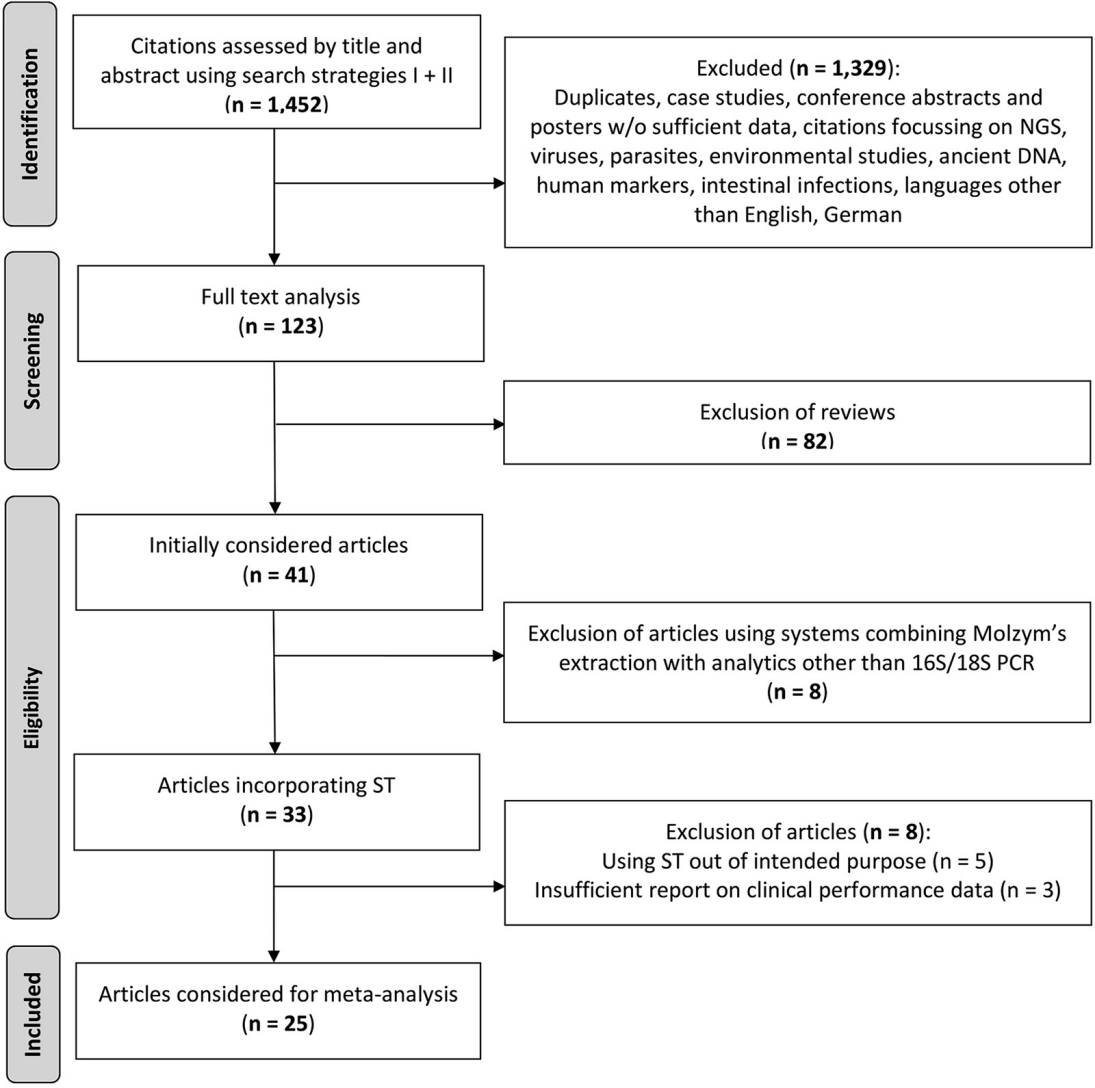
Study selection procedure following PRISMA-P guidelines.

The literature search resulted in 1,452 citations, of which, after removal of duplicate and irrelevant citations, 123 were considered for full text analysis (Fig. 1). After exclusion of reviews and papers that involved other devices, did not follow the advised technical procedure, used ST outside the intended purpose, or lacked a characterization of the cultural state of samples to allow accuracy and added-value calculations, 25 eligible references remained.

### Study characteristics and patient populations.

The 25 studies included 4,646 samples from 3,351 patients. Twenty studies reported the performance of ST related to samples (4,269 in total) and 5 studies to patients (185 in total), thus ending up with a sample size of 4,454 (Table 1). Eighteen studies focused on single diseases, and 7 employed patient populations of various diseases (VD) from routine diagnosis. Most data of the latter could not be retroactively attributed to specific diseases and were hence analyzed as a heterogeneous disease group. In summary, 9 studies focused on sepsis (SP), 7 on VD, 5 on infectious endocarditis (IE), 2 on bacterial meningitis (BM), 1 on joint infections (JI), and 1 on pyomyositis (PM). A total of 22 studies compared ST to culture or Duke-classified patients (IE) serving as the standard references (the studies are further characterized and analyzed in “Study quality” and “Clinical study performance” below). The remaining 3 studies (41, 42, 44) (Table 1) used samples from culture-negative patients to determine the incidence of nongrowing pathogens. Eighteen studies were prospective, among them 2 multicenter studies, 4 retrospective, and 3 unspecified (Table 1). Nineteen studies were observational, 3 interventional, and 3 unspecified. Twenty-four studies were conducted in Europe and one in India. Patient populations were recruited from multiple (10/25 studies) or single (10/25) wards (Table 1). In 5 studies, no information on the origin of patient populations was provided.

**TABLE 1:** Characteristics of the 25 studies included^*a*^

Disease (no. of studies), reference	Sample size	Country(ies) of study^*a*^	Specimen type(s)^*b*^	Type of study	Study aim(s)	Patient population(s)^*c*^
Sepsis (*n* = 9)						
21	342	DE	Blood	Prospective, observational, multicenter	Rapid detection; detection of fastidious/noncultivable pathogens	SIRS, immunodeficiency, in ICU, hematology, oncology
22	20	DE	Swabs of membrane oxygenators	Prospective, observational	Rapid detection; early antibiotic adjustment; detection of fastidious/noncultivable pathogens	Application of antibiotics before sampling, in surgery, transplantation, thorax/heart intervention
23	75	AT	Blood	Prospective, observational	Rapid detection; early antibiotic adjustment; detection of fastidious/noncultivable pathogens	Critically ill, not further specified, 46% of patients received antibiotics before sampling, in unspecified wards
24	50	DE	Blood	Prospective, observational	Rapid detection; early antibiotic adjustment	Adults >18 years, signs of sepsis, in ICU
25	160	DE	Blood	Prospective, observational	Rapid detection; detection of fastidious/noncultivable pathogens	Application of antibiotics before sampling, in surgery, transplantation, thorax/heart intervention
26	23	SI	Blood	Prospective, observational	Rapid detection; early antibiotic adjustment	SIRS, application of antibiotics before sampling, in ED
27	236	DE, NL	Blood	Prospective, observational multicenter	Rapid detection; early antibiotic adjustment; detection of fastidious/noncultivable pathogens	Adults >18 years, in ICU
28	9	IN	Blood	Unspecified	Applicability as diagnostic for routine	Surgery, heart transplantation
29	160	CZ	Blood	Prospective, observational	Applicability as diagnostic for routine	Hospitalized patients with suspected BSI, in ICU

Infectious endocarditis (*n* = 5)						
30	30	DE	Heart valves	Prospective, observational	Rapid detection; detection of broad range of pathogens	Surgery, heart intervention
31	46	DE	Heart valves	Prospective, interventional	Rapid detection; early antibiotic adjustment	Surgery, heart intervention
32	40	BE	Heart valves	retrospective, observational	Rapid detection; detection of broad range of pathogens	Surgery, heart intervention
33	127	BE	Heart valves	Prospective, interventional	Applicability as diagnostic for routine; early antibiotic adjustment	Surgery, heart intervention
34	8	FR	Heart valves	Retrospective, observational	Applicability as diagnostic for routine; early antibiotic adjustment	Low concentration of CRP, in ICU

Joint infections (*n* = 1)						
35	54	DE	Bones, soft tissues	Prospective, observational	Detection of broad range of pathogens	Septic/aseptic, exchange/revision of joint prostheses, in surgery, traumatology

Bacterial meningitis (*n* = 2)						
36	40	DE	CSF	Unspecified	Detection of broad range of pathogens	Adults, children in university hospital in unspecified wards
37	38	SI	CSF	Prospective, observational multicenter	Applicability as diagnostic for routine	In ICU, surgery, pediatrics
						
Pyomyositis (*n* = 1)						
38	12	FR	Pus of muscles	Unspecified	Applicability as diagnostic for routine	HIV infection, cancer, cirrhosis, infectious endocarditis, other, in infectiology, hemodialysis

Various diseases (*n* = 7)^*d*^						
39	104	AT	Aspirates, tissues	Prospective, observational	Applicability as diagnostic for routine	In orthopedics, other unspecified departments in university hospital, tertiary hospital, private physicians
40	66	DE	Blood, aspirates, tissues, swabs, rinse fluids	Retrospective, observational	Applicability as diagnostic for routine	In orthopedics, cardiology, general hospital
41	76	DK	Bones, tissues, aspirates, implants, heart valves, pus, rinse fluids, swabs	Prospective, observational	Detection of fastidious/noncultivable pathogens	Culture-negative patients with suspicion of an infection, in secondary and tertiary hospitals in unspecified wards
15	1370	CZ	Blood, aspirates, tissues	Prospective, observational	Detection of fastidious/noncultivable pathogens	Pediatric, in hematology, anesthesiology, oncology, ICU, cardiovascular surgery
42	529	DK	Aspirates, tissues, BAL fluids, rinse fluids	Retrospective, interventional	Early antibiotic adjustment	Culture-negative patients with suspicion of an infection, in secondary and tertiary hospitals
43	772	CH	Aspirates, tissues, swabs, rinse fluids, secretions, other	Prospective, observational	Rapid detection; applicability as diagnostic for routine; detection of fastidious/noncultivable pathogens	In unspecified wards
44	67	FR	Various tissue and fluid samples	Prospective, observational	Applicability as diagnostic; routine detection of fastidious/noncultivable pathogens	Culture-negative patients with suspect of an infection, in unspecified wards

a AT, Austria; BE, Belgium; CH, Switzerland; CZ, Czech Republic; DE, Germany; DK, Denmark; FR, France; IN, India; NL, The Netherlands; SI, Slovenia.

b CSF, cerebrospinal fluid; BAL, bronchoalveolar lavage.

c SIRS, systemic inflammatory response syndrome; ICU, intensive care unit; ED, emergency department; BSI, bloodstream infection; CRP, C-reactive protein.

d Various diseases from routine diagnosis, including infections of joints, bones and soft tissues, abscesses, infectious endocarditis, pericarditis, bacterial meningitis, pleuritis, hematomas, septic arthritis, peritonitis, adnexitis, genitourinary infections, and others.

### Study aims of publications.

Various ambitions prompted the authors of the studies to evaluate ST. In the majority of studies (14/25), more than one aim was expressed (Table 1). An often-declared aim was to find out the suitability of ST as a supplement to culture for routine diagnosis (SP, 2/9 studies; IE, 2/5; BM, 1/2; VD, 4/7; PM, 1/1). More specifically, rapid detection of pathogens was a reason to use ST for SP (7/9 studies) and IE (3/5), although only two studies estimated time to results. Wellinghausen et al. (21) measured 19.2 h for the mean time to positive signaling for culturing compared with 7 to 8 h or overnight by the ST workflow. Orszag et al. (25) reported availability of ST results in 45% of cases 13 to 75 h earlier than for culture. Another reported reason for evaluation of ST included its impact on early implementation of patient treatment, including initiation, change, or stoppage of the application of antibiotics (SP, 5/9 studies; IE, 3/5; VD, 1/7). Further declared aims were the detection of fastidious and noncultivable pathogens in populations with SP (5/9 studies) and VD (4/7), as well as the analysis of pathogens in culture-negative samples from VD patients (3/7 studies). Finally, the potential capacity of detection of a broad range of pathogens was an argument to test ST with IE (2/5 studies), BM (1/2), and JI (1/1).

### Study methodologies.

Studies analyzed bacteria and fungi (12/25 studies) or bacteria only (12/25). One study (23) did not indicate whether, in addition to the 16S rRNA gene assay, the 18S rRNA gene assay was also run. Among 21 studies, reference methods followed standard practices of culturing in liquid and on agar media or modified Duke criteria for the classification of IE. Three studies used culture-negative samples exclusively, and one study did not provide specific information on culturing methods.

In 9 of 25 studies, the interpretation of ST results with culture-negative samples followed a specified algorithm of evidence of infection or contamination (21). Accordingly, results were assigned as “probably true” infections if the organisms identified were supported by the history of the patient’s disease, signs of infection in clinical imaging, elevated levels of inflammatory markers, consensus with other same-day or earlier cultivated materials, and/or molecular tests. “Possibly true” results were assigned for cases where common disease pathogens were identified without further culture results. “Signs of contamination” were assumed if a nonpathogenic organism or an organism without known relation to the disease was detected. In other studies (15/25 studies), ST results were interpreted by using a constructed comparator (e.g., referring to results from previous and current blood cultures [23]) or by clinicians without specifications mentioned. One study did not provide information on the clinical evaluation.

### Study quality.

**FIG 2:**
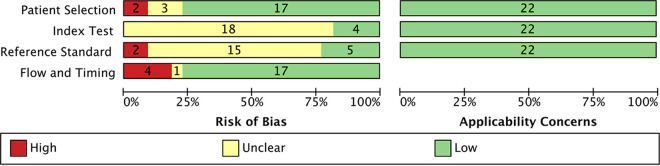
Evaluation of quality of included studies using QUADAS-2.

In total, 22 studies using reference standards were validated with respect to quality and bias according to QUADAS-2 criteria (Fig. 2). In all but one study (36), a case-control design was avoided. Patient selection bore a low risk of bias in 17 of 22 studies (77%). A high risk of bias and some concern were noted for 2 and 3 studies (23%), respectively, mostly associated with doubts of whether consecutive or random samples of patients were enrolled. Only 4 studies (18%) provided information that the index test results were interpreted without knowledge of reference results. With the remaining 18 studies, this issue was unclear. In 5 studies (23%), reference results were interpreted without knowledge of the index test, while 2 studies left bias (9%) and 15 (68%) concerns about independent interpretation. Finally, flow and timing between the reference standard and index test were followed by 17 of 22 studies (77%). Bias and some concern were noted in 4 (18%) and 1 (4%) study, respectively. Here, doubts were raised for all three questions regarding whether an appropriate interval between index test and reference standard existed, patients received the same reference standard, and all patients were analyzed. There were no applicability concerns in any study.

### Accuracy.

**FIG 3:**
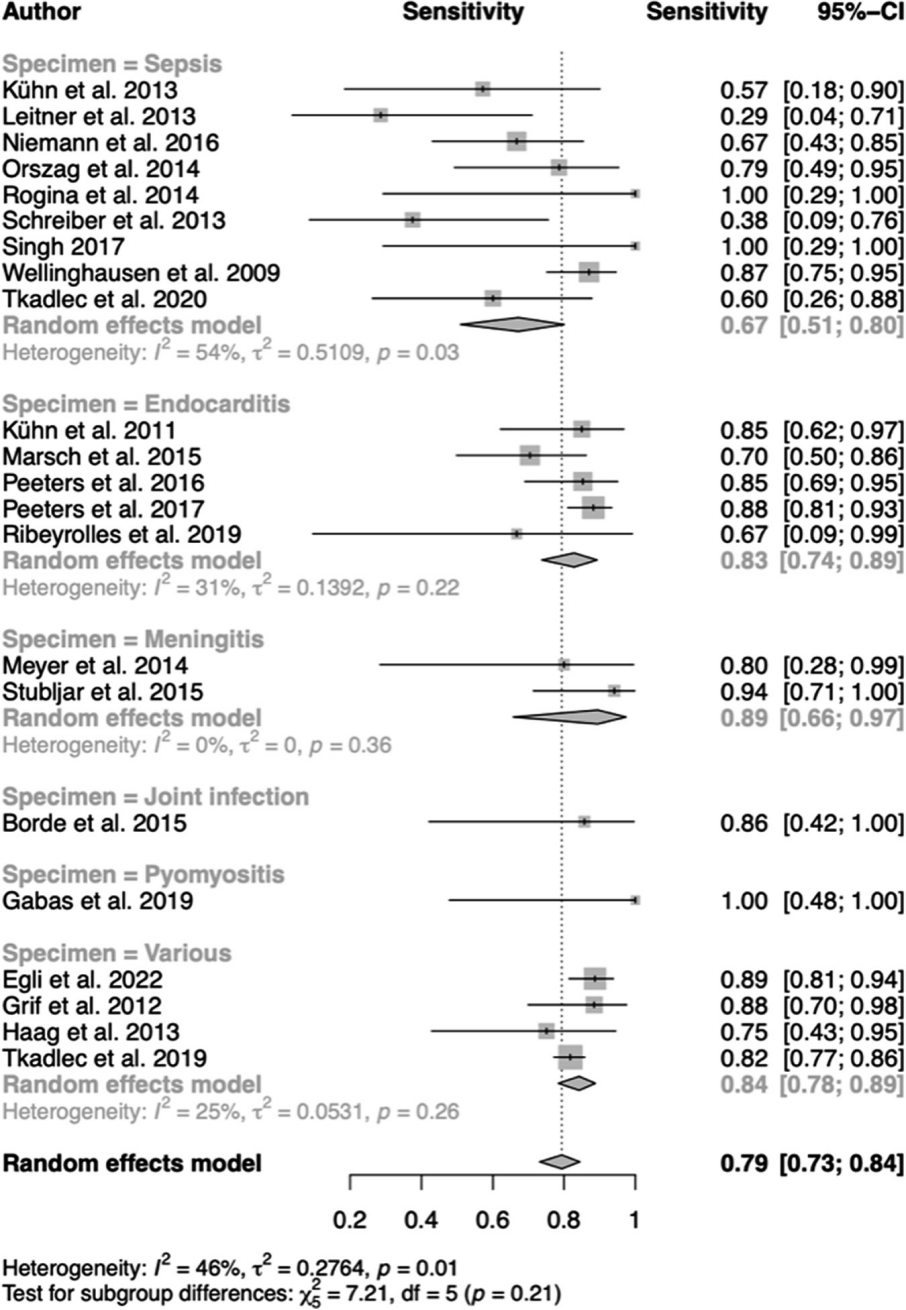
Forest plots of pooled and individual diagnostic sensitivities of disease subgroups.

The pooled sensitivity of ST across diseases was 79% (95% confidence interval [CI], 73 to 84%), with large effect sizes (67 to 100%) and heterogeneity near the “substantial” threshold (*I*^2^ = 46%, *P* = 0.01) (Fig. 3). Among the disease subgroups with at least two studies, BM showed the highest sensitivity (89%; 95% CI, 66 to 97%), followed by VD (84%; 95% CI, 78 to 89%), IE (83%; 95% CI, 74 to 89%), and SP (67%; 95% CI, 51 to 80%). While heterogeneity was substantial with SP (*I*^2^ = 54%; *P* = 0.03), subgroups BM, VD, and IE uncovered low to some heterogeneity. In the subgroups and the pooled estimate, the sensitivity of ST was mainly due to random distribution.

**FIG 4:**
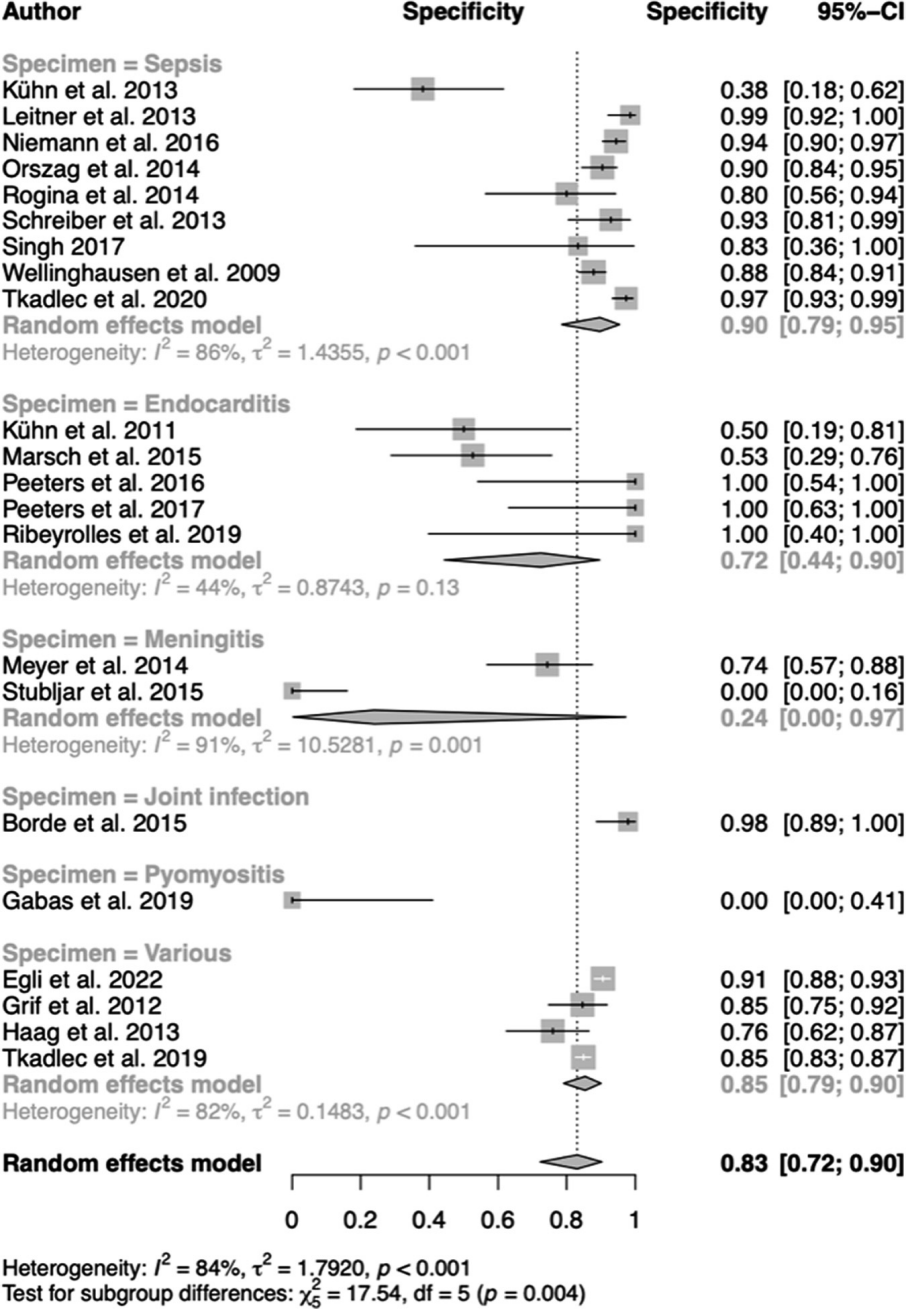
Forest plots of pooled and individual diagnostic specificities of disease subgroups.

The pooled diagnostic specificity of ST (83%; 95% CI, 72 to 90%) exhibited high effect sizes (24 to 90%) combined with considerable heterogeneity (*I*^2^ = 82%, *P* < 0.001) (Fig. 4). The highest specificity was found with sepsis (90%; 95% CI, 79 to 95%), followed by VD (85%; 95% CI, 79 to 90%), IE (72%; 95% CI, 44 to 90%), and BM (24%; 95% CI, 0 to 97%). Heterogeneity was substantial among all subgroups.

**FIG 5:**
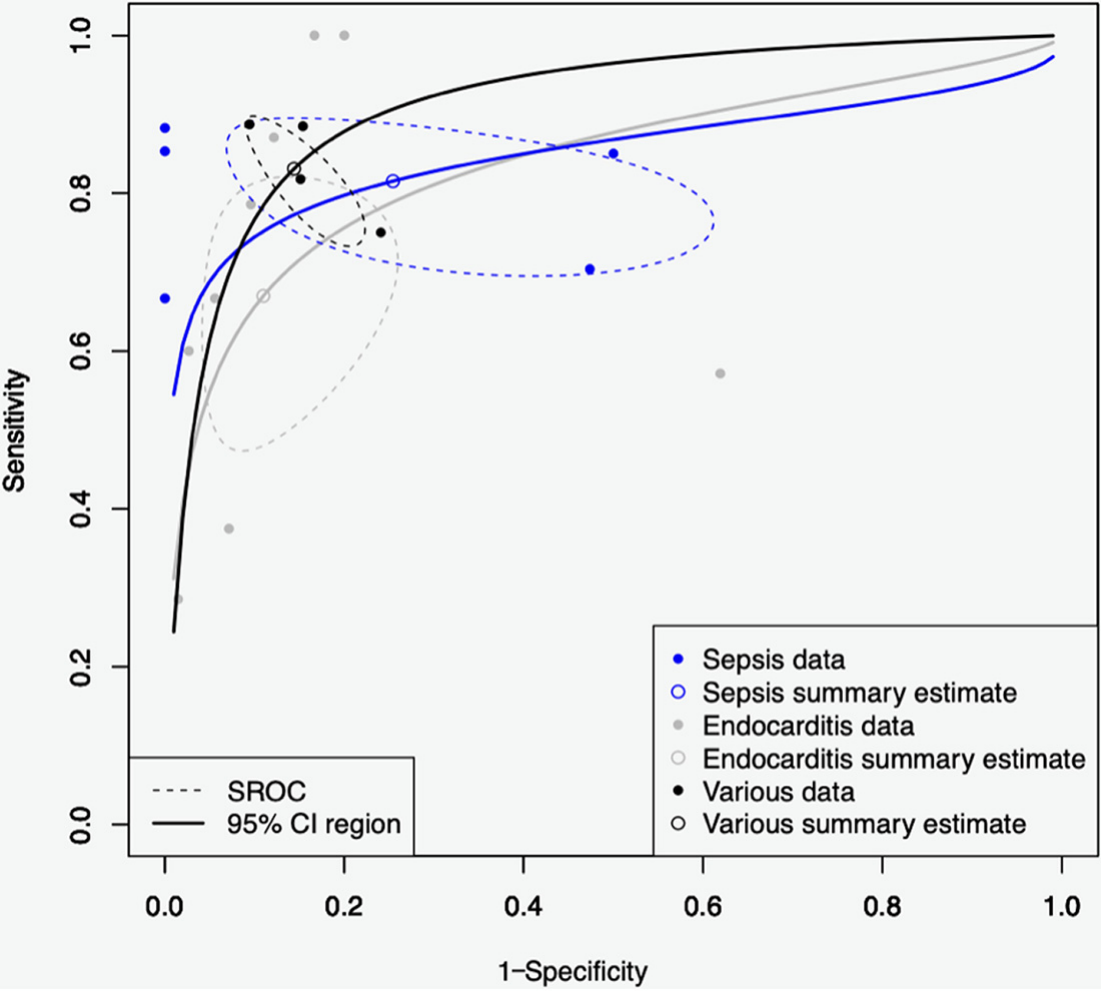
Summary receiver operating characteristic (SROC) curve (bivariate model) for diagnostic test accuracy of subgroups SP, IE, and VD. Sensitivity and specificity data of the individual studies are represented by full dots and summary estimates of sensitivity and specificity by open dots in the curves. The dotted ovals represent the 95% confidence regions for the summary estimates.

Heterogeneity in the estimates of sensitivity and specificity across subgroups of diseases can lead to heterogeneity in thresholds. For each subgroup with at least 3 studies, separate estimates of both characters were plotted against each other to calculate summary ROC (SROC) curves and areas under the curves (Fig. 5). For SP, the pooled estimates of sensitivity and specificity were 67% and 81%, respectively, with an area under the curve of 0.83. For IE, the pooled estimates of sensitivity and specificity were 82% and 75%, respectively (area, 0.84). Finally, the sensitivity and specificity values of VD were 83% and 86%, respectively (area, 0.83). The areas under the curves indicate an acceptable diagnostic accuracy of ST as regards the three main subgroups.

**FIG 6:**
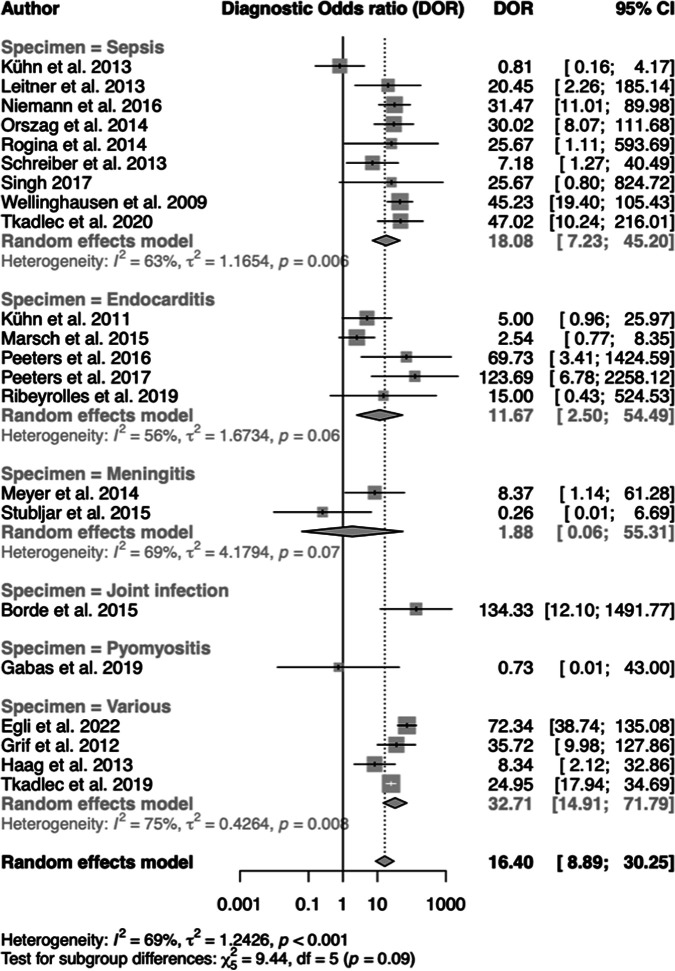
Forest plots of diagnostic odds ratios across subgroups of diseases.

The pooled diagnostic odds ratio of the random effect model across all studies was 16.4 (95% CI, 8.89 to 30.25%) (Fig. 6). Higgins’ *I*^2^ of 69% (*P* < 0.001) confirmed substantial heterogeneity across all studies. Subgroup differences were not significant (*P* = 0.09). Overall, this statistical test indicated that ST presented as a correctly discriminating diagnostic procedure, particularly in multiple-study groups SP, IE, and VD.

### Added value.

**FIG 7:**
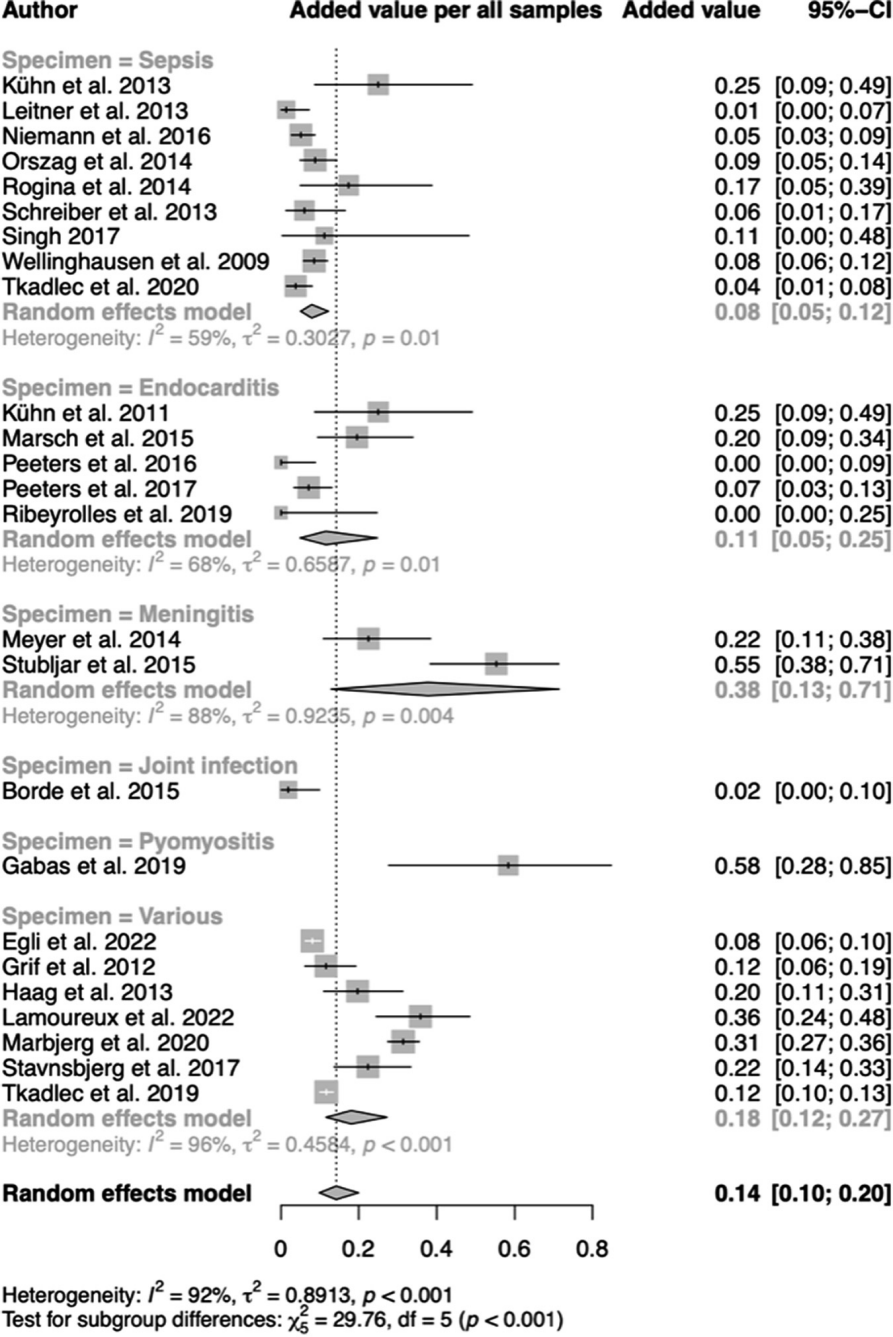
Forest plots of pooled added value across subgroups of diseases.

Tkadlec et al. (15) termed relevant microorganisms found by ST in culture-negative samples as being of “added value” to reassure clinicians in starting, adjusting, or continuing antibiotic treatment. Following this definition for all studies and adding de-escalation of antibiotic treatment as added value (33), the pooled analysis indicated 14% (95% CI, 10 to 20%) added value in the total sample size (Fig. 7). The added value varied considerably among subgroups (2 to 58%) and showed high heterogeneity in the pooled analysis (*I*^2^ = 92%, *P* < 0.001).

### Clinical study performance.

Among the 22 studies, the overall positive rate of bacteria and fungi determined by ST was 34% (95% CI, 33 to 36%), and thus, significantly higher (*P < *0.001) than that of culture, which was 20% (95% CI, 19 to 21%) (not shown). False-positive rates of culture and ST in terms of contaminants judged by the authors of the papers were determined on the basis of sample sizes of 766 (11 studies) and 3,000 (20 studies), respectively. As a result, the rates of culture (7%; 95% CI, 5 to 9%) and ST (8%; 95% CI, 7 to 9%) were not significantly different (*P* = 0.352). The false-negative rate of ST (21 studies, 3,772 samples) was 4% (95% CI, 3 to 5%), and thus, significantly lower (*P < *0.001) than that of culture (6 studies, 413 samples), at 15% (95% CI, 11 to 18%). Technical failure of ST, including PCR inhibition or failure of sequencing, was reported by 9 studies with 1,672 samples in total (not shown). The failure rates of the 3 platforms were <0.6 to 3.1% (SepsiTest, *n* = 6), 0.6 and 4.5% (UMD-SelectNA, *n* = 2), and 0.3% (Micro-Dx, *n* = 1). The accumulated rate across the platforms was 0.6% (95% CI, 0.3 to 1.1%).

### Changes in antibiotic treatment.

Among 4 studies encompassing 432 patients (31, 33, 34, 42), 51 patients (12% [95% CI, 9 to 15%]) from the IE (3 studies) and VD (1 study) groups were subjected to changes of antibiotic therapy upon availability of ST results (not shown). Antibiotic therapy was started for 3, narrowed for 43, or stopped for 5 patients. For instance, in a culture-negative IE case, ST identified Coxiella burnetii, which caused the initiation of antibiotic therapy with doxycycline and hydrochloroquine (see Table S4 of reference 33). In another IE patient, blood culture and valve culture disagreed by identifying Staphylococcus epidermidis and Cutibacterium acnes, respectively. ST confirmed the valve culture result, upon which therapy was switched from vancomycin and rifampin to amoxicillin and rifampin. Finally, antibiotic therapy of culture-negative definitive or possible IE was stopped when ST results were also negative (33).

### Incidence of clinically relevant bacterial groups.

Bacteria found by culture and ST were sorted into clinically relevant groups (15). Accordingly, the frequencies of (i) Gram-positive bacteria with subgroups coagulase-negative staphylococci (CoNS), Staphylococcus aureus, streptococci, and enterococci, (ii) Gram-negative bacteria, including *Enterobacteriaceae* and nonfermenting Gram-negative bacilli, and (iii) rare, fastidious, and anaerobic bacteria were determined (Table 2). The frequency of a particular bacterial group was calculated only from studies where it had actually been detected. Data were extracted from 3,002 cultured samples (20 studies) and 3,577 samples analyzed by ST (22 studies). Across all disease subgroups, culture was positive for bacteria in 613 samples, or 20.4% (95% CI, 18.8 to 22.1%), and ST in 1,152 samples, or 32.2% (95% CI, 30.4 to 34.1%). This difference was statistically significant (*P < *0.001).

**TABLE 2:** Frequencies of clinically grouped pathogens found in disease subgroups^*a*^

Pathogen group^*a*^	Value [no. of samples or patients (no. of studies), mean % (95% CI), or *P* value] for indicated disease subgroup and test^*b*^																	
	Sepsis			Infectious endocarditis			Various diseases			Bacterial meningitis			Joint infection			Pyomyositis		
	Culture	ST	*P* value	Culture	ST	*P* value	Culture	ST	*P* value	Culture	ST	*P* value	Culture	ST	*P* value	Culture	ST	*P* value
Bacteria from all groups																		
Samples (studies)	1,075 (9)	1,075 (9)		205 (4)	251 (5)		1,616 (4)	2,145 (5)		40 (1)	40 (1)		54 (1)	54 (1)		12 (1)	12 (1)	
Positivity	13.8 (11.6–16.2)	20.2 (17.6–23.1)	0.116	27.8 (21.1–36.0)	83.7 (72.7–95.8)	**<0.001**	24.2 (21.9–26.7)	32.4 (30.0–34.9)	**<0.001**	12.5 (4.1–29.2)	32.5 (17.3–55.6)	**0.033**	13.0 (5.2–26.7)	11.1 (4.1–24.2)	0.763	41.7 (13.5–97.2)	100 (54.7–100)	**0.002**

Gram positives																		
Samples, all (studies)	1,066 (8)	1,066 (8)		197 (3)	243 (4)		1,616 (4)	2,145 (5)		40 (1)	40 (1)		54 (1)	54 (1)		12 (1)	12 (1)	
Positivity, all	7.8 (6.2–9.7)	10.1 (9.0–13.1)	0.063	21.3 (15.4–28.8)	68.3 (58.3–79.5)	**<0.001**	15.5 (13.7–17.6)	21.1 (19.2–23.1)	**<0.001**	5.0 (0.6–18.1)	17.5 (7.0–36.1)	0.079	11.1 (4.1–24.2)	9.3 (3.0–21.6)	0.758	16.7 (2.0–60.2)	66.7 (28.8–100.0)	**0.0150**

CoNS																		
Samples (studies)	883 (6)	883 (6)		157 (2)	243 (4)		1,616 (4)	2,145 (5)		40 (1)	40 (1)		54 (1)	54 (1)		12 (1)	12 (1)	
Positivity	4.5 (3.2–6.2)	6.2 (4.7–8.1)	0.113	4.5 (1.8–9.2)	11.5 (7.7–16.7)	**0.016**	6.9 (5.7–8.3)	7.6 (6.4–8.8)	0.414	ND	ND		7.4 (2.0–19.0)	5.6 (1.1–16.2)	0.706	ND	ND	

Staphylococcus aureus																		
Samples (studies)	1,066 (8)	1,066 (8)		167 (2)	197 (3)		1,616 (4)	2,145 (5)		40 (1)	40 (1)		54 (1)	54 (1)		12 (1)	12 (1)	
Positivity	1.7 (1.0–2.7)	2.3 (1.5–3.5)	0.323	6.6 (3.3–11.8)	17.8 (12.4–24.7)	**0.001**	4.6 (3.7–5.8)	6.6 (5.6–7.8)	**0.009**	ND	ND		1.8 (0.0–10.3)	1.8 (0.0–10.3)	>0.999	8.3 (0.2–46.4)	33.3 (9.1–85.4)	0.140

Streptococci																		
Samples (studies)	572 (4)	1,066 (8)		167 (2)	243 (4)		1,550 (3)	2,145 (5)		40 (1)	40 (1)		54 (1)	54 (1)		12 (1)	12 (1)	
Positivity	1.7 (0.8–3.2)	2.0 (1.2–3.0)	0.671	4.2 (1.7–8.6)	30.4 (23.9–38.2)	**<0.001**	2.2 (1.6–3.1)	5.4 (4.5–6.5)	**<0.001**	5.0 (0.6–18.1)	17.5 (7.0–36.1)	0.079	ND	ND		ND	25.0 (5.2–76.1)	

Enterococci																		
Samples (studies)	626 (5)	918 (5)		197 (3)	243 (4)		1,540 (3)	2,069 (4)		40 (1)	40 (1)		54 (1)	54 (1)		12 (1)	12 (1)	
Positivity	2.4 (1.3–4.0)	1.6 (1.6–2.7)	0.261	8.6 (5.0–13.8)	11.9 (8.0–17.1)	0.2605	1.9 (1.3–2.8)	1.5 (1.1–2.2)	0.354	ND	ND		1.8 (0.0–10.3)	1.8 (0.0–10.3)	>0.999	8.3 (0.2–46.4)	8.3 (0.2–46.4)	>0.999

Gram negatives																		
Samples, all (studies)	1,075 (9)	1,075 (9)		127 (1)	157 (2)		1,474 (2)	2,069 (4)		40 (1)	40 (1)		54 (1)	54 (1)		12 (1)	12 (1)	
Positivity, all	2.8 (1.9–4.0)	3.6 (2.6–5.0)	0.292	1.6 (0.2–5.6)	1.9 (0.4–5.6)	0.849	6.5 (5.3–8.0)	4.6 (3.8–5.7)	**0.014**	5.0 (0.6–18.1)	7.5 (1.5–21.9)	0.646	1.8 (0.0–10.3)	1.8 (0.0–10.3)	>0.999	8.3 (0.2–46.4)	8.3 (0.2–46.4)	>0.999

*Enterobacteriaceae*																		
Samples (studies)	1,075 (9)	1,075 (9)		127 (1)	157 (2)		1,474 (2)	2,069 (4)		40 (1)	40 (1)		54 (1)	54 (1)		12 (1)	12 (1)	
Positivity	2.4 (1.6–3.5)	2.7 (1.8–3.9)	0.659	0.8 (0.0–4.4)	1.2 (0.2–4.6)	0.739	5.0 (3.9–6.3)	3.6 (2.8–4.5)	**0.040**	2.5 (0.1–13.9)	ND		ND	ND		8.3 (0.2–46.4)	8.3 (0.2–46.4)	>0.999

Nonfermenting Gram-negative bacilli																		
Samples (studies)	511 (3)	747 (4)		127 (1)	127 (1)		1,474 (2)	2,003 (3)		40 (1)	40 (1)		54 (1)	54 (1)		12 (1)	12 (1)	
Positivity	0.8 (0.2–2.0)	1.3 (0.6–2.0)	0.403	0.8 (0.0–4.4)	0.8 (0.0–4.4)	>0.999	1.4 (0.9–2.3)	1.1 (0.7–1.7)	0.427	2.5 (0.1–13.9)	7.5 (1.5–21.9)	0.308	1.8 (0.0–10.3)	1.8 (0.0–10.3)	>0.999	ND	ND	

Rare, fastidious, and anaerobic bacteria																		
Samples (studies)	577 (3)	918 (5)		24 (2)	251 (5)		1,550 (3)	2,145 (5)		40 (1)	40 (1)		54 (1)	54 (1)		12 (1)	12 (1)	
Positivity	0.9 (0.3–2.0)	2.9 (1.9–4.3)	**0.007**	8.3 (1.0–30.1)	9.1 (5.8–13.8)	0.896	1.7 (1.1–2.5)	5.6 (4.6–6.7)	**<0.001**	2.5 (0.1–13.9)	7.5 (1.5–21.9)	0.308	ND	ND		16.7 (2.0–60.2)	25.0 (5.2–76.1)	0.624

a For genera and species detected in the groups, see Table 3. CoNS, coagulase-negative staphylococci.

b Numbers of samples or patients and numbers of studies where the pathogens were detected based on the calculation of positivity are given. Bacteria in mixed infections were included as single counts. *P* values in boldface are significant. ND, not detected.

Among the subgroups, the positivity of bacteria by culture and ST ranged from 12.5 to 41.7% and 11.1% to 100%, respectively (Table 2). In IE, VD, BM, and PM, the positivity of ST was significantly higher than that of culture. In particular, ST detected pathogens at frequencies that added 34% (VD), 240% (PM), 260% (BM), and 300% (IE) to the frequencies determined by culture.

Both culture and ST generally agreed in the finding that Gram positives formed the most abundant group, with positivity rates ranging from 8 to 21% (5/6 subgroups) and 9 to 68% (6/6 subgroups), respectively (Table 2). In IE and VD, ST detected significantly more S. aureus and streptococci than culture. Furthermore, in IE, ST also found more CoNS than did culture. There was no significant difference in positivity between ST (range, 1.8 to 8.3%) and culture (1.6 to 8.3%) in the detection of enterococci and Gram-negative pathogens, except in VD (Table 2). Here, culture grew significantly more Gram negatives, in particular *Enterobacteriaceae*, than occurred in ST (5.0% versus 3.6%). Rare, fastidious, and anaerobic bacteria formed a surprisingly abundant group of pathogens, with positivity rates ranging from 0.9 to 16.7% and 2.9 to 25.0% by culture and ST, respectively. Significant differences between culture and ST were observed in SP (0.9 versus 2.9%) and VD (1.7 versus 5.6%).

### Diversity of ST-identified pathogens.

In total, 127 bacterial taxa (44 at the genus and 83 at the species level) and 3 fungal taxa were identified by ST (Table 3). With 54 taxa, the bacterial group “rare, fastidious, and anaerobic bacteria” appeared as the most diverse. For comparison, culture identified only 11 taxa (not shown). Furthermore, nonfermenting Gram-negative bacilli were represented by Pseudomonas aeruginosa according to culture (not shown), while ST found another 15 bacterial taxa in addition. Bacteria in the other groups, as well as fungal taxa, were common pathogens also represented by similar numbers of taxa in culture (not shown). In summary, ST identified a high diversity of bacterial taxa, which to a great extent came from the clinical groups “rare, fastidious, and anaerobic bacteria” and “nonfermenting Gram-negative bacilli.”

**TABLE 3:** Clinically relevant microorganisms identified by ST

Bacterial group (no. of taxa)	Taxon
Staphylococci (*n* = 15)	Staphylococcus spp., S. arlettae, S. aureus, S. auricularis, S. capitis, S. cohni, S. epidermidis, S. gallinarum, S. haemolyticus, S. hominis, S. lugdunensis, S. pettenkoferi, S. saccharolyticus, S. sciuri, S. warneri
Streptococci (*n* = 23)	Abiotrophia defectiva, Granulicatella adiacens, G. elegans, Streptococcus spp., S. agalactiae, S. anginosus, S. bovis, S. capitis, S. constellatus, S. cristatus, S. dysgalactiae, S. equi, S. gallolyticus, S. gordonii, S. milleri, S. mitis, S. mutans, S. oralis, S. pneumoniae, S. pyogenes, S. sanguinis, S. salivarius, S. viridans
Enterococci (*n* = 7)	Enterococcus spp., E. casseliflavus, E. cecorum, E. faecalis, E. faecium, E. gallinarum, E. mundtii
*Enterobacteriaceae* (*n* = 12)	Citrobacter spp., Enterobacter spp., Enterobacter aerogenes, Escherichia coli, Klebsiella spp., Klebsiella oxytoca, Klebsiella pneumoniae, Proteus spp., Proteus mirabilis, Providencia spp., Serratia spp., Serratia marcescens
Nonfermenting Gram-negative bacilli (*n* = 16)	Acinetobacter spp., Acinetobacter baumannii, Acinetobacter lwoffii, Achromobacter spp., Burkholderia spp., Edwardsiella spp., Edwardsiella ictaluri, Moraxella spp., Pseudomonas spp., Pseudomonas aeruginosa, Pseudomonas extremorientalis, Pseudomonas otitidis, Paracoccus spp., Sphingomonas spp., Stenotrophomonas spp., S. maltophilia
Rare, fastidious, and anaerobic bacteria (*n* = 54)	Aerococcus spp., Aggregatibacter spp., Aggregatibacter actinomycetemcomitans, Anaerococcus spp., Arcanobacterium haemolyticum, Bacillus spp., Bacillus siralis, Bacteroides spp., Bacteroides fragilis, Bartonella henselae, Borrelia spp., Capnocytophaga canimorsus, Cardiobacterium hominis, Clostridium spp., Corynebacterium spp., Corynebacterium amycolatum, Corynebacterium jeikeium, Corynebacterium pseudotuberculosis, Coxiella burnetii, Cutibacterium spp., Cutibacterium acnes, Dialister pneumosintes, Facklamia languida, Finegoldia magna, Fusobacterium spp., Fusobacterium nucleatum, Gemella bergeri, Haemophilus spp., Haemophilus influenzae, Haemophilus parainfluenzae, Kocuria spp., Lactobacillus spp., Lactococcus spp., Legionella spp., L. pneumophila, Leptotrichia spp., Listeria monocytogenes, Massilia spp., Micrococcus spp., Micrococcus luteus, Mycobacterium spp., Mycobacterium tuberculosis, Mycoplasma hominis, Neisseria meningitidis, Nocardioides spp., Parvimonas micra, Peptostreptococcus spp., Raoultella spp., Rhodococcus spp., Rothia spp., Tropheryma whipplei, Turicibacter spp., Weissella spp., Ureaplasma urealyticum
Fungi (*n* = 3)	Aspergillus fumigatus, Candida albicans, Candida glabrata

## APPLICABILITY OF SEPSITEST PLATFORM IN CLINICAL USE

The clinical performance of the CE-approved 16S and 18S rRNA gene Sanger sequencing system SepsiTest (ST) was analyzed based on 25 eligible studies employing clinical specimens without cultivation. It is noteworthy that the method eliminates free DNA and, thus, analyzes only sequences from living microorganisms. The ST system seems to be robust in terms of technical performance. The rate of failure among the three ST platforms (0.3 to 4.5%) compares with those in other diagnostic systems, including the Roche SeptiFast (1.5 to 10.8%) (45–48), T2Biosystems T2Bacteria (2.8%) (49), and Abbott Iridica (4.0%) (50).

Sepsis formed the largest disease subgroup and exhibited the lowest sensitivity among the diseases analyzed (Fig. 3). Low sensitivity may be attributed to sampling errors because of the employment of considerably higher blood volumes in culture (typically, 40 to 60 mL in adults) than in ST (up to 10 mL) (27). This and low loads of bacteria in the blood (1 to 30 CFU/mL) (51, 52) that were at or below the detection limits of ST (20 to 80 CFU/mL) may have caused negative results with culture-positive samples (22). Interestingly, the estimated sensitivity (67%, 95% CI, 51 to 80%) was comparable to that of a syndromic real-time PCR panel assay, Roche SeptiFast (68%, 95% CI, 63 to 73%) (53), and a species-agnostic Abbott PCR-electrospray ionization mass spectrometry (ESI-MS) system, the Iridica (66%, 95% CI, 57 to 74%) (54).

It is of note that the issue of low ST sensitivity for pathogens in blood appears to have resulted in a lower detection rate of *Enterobacteriaceae* than in culture in the case of VD-related clinical materials (Table 2) that had been represented mostly by blood samples (15). *Enterobacteriaceae*, in general, grow well, and it has already been shown that they have the highest chance of all of the groups of pathogens to be detected by culture (15). On the other hand, in the sepsis disease subgroup, the detection rates of *Enterobacteriaceae* were comparable between culture and ST (Table 2). Whether the conflicting results of *Enterobacteriaceae* detection by ST in blood in the two disease subgroups is a matter of heterogeneity, i.e., the impacts of different study designs, populations, and study qualities, or a real phenomenon is an issue that has to be further addressed.

Regarding an impact on clinical decision-making for de-escalation of antibiotic treatment of patients, the estimated overall specificity of ST (83%) (Fig. 4) is of limited value. Limited specificity is caused by microorganisms that are found in addition to culture. In this review, eligible studies employing ST point out a clear trend toward the recognition of the importance of microorganisms with judged clinical relevance found in addition to culture (Fig. 7). Consequently, three recent studies analyzed ST-positive, culture-negative samples only (Table 2). A benefit of this so-called “added value” (15), i.e., the detection of nongrowing pathogens by ST, is important in view of initiating antibiotic treatment or changing to targeted therapy. However, only 4 of the 25 studies presented data that point at a potentially considerable contribution of ST results to a change of the treatment. Clearly, future intervention studies should focus on the consequences of ST-positive, culture-negative results regarding treatment of patients presenting with clear signs of infection.

A particular advantage of an agnostic approach like ST is the identification of infections caused by anaerobes and other fastidious species (42). This view is confirmed by this review. The uncovering of a great variety of taxa within these groups emphasizes the strategy of combined deployment of culture and ST, which may contribute to a more comprehensive diagnosis of the infectious status of ill patients. This, however, increases the costs of diagnosis: estimates of the costs of ST usage range from 108 to 149 GBP (approximately $128 to $177) for reagents, manual working time, equipment, and overhead, depending on medium or low throughput (55). These added costs might be put into perspective against the background in potential added value in diagnosing nongrowing pathogens and adjustments of antibiotic treatment of ill patients.

Sanger-based diagnosis is now usually perceived as a predecessor of metagenomics, another species-agnostic molecular approach that utilizes next-generation sequencing (NGS) technologies. Indeed, this alternative offers advantages over Sanger-based ST, such as easier detection of multiple microorganisms (i.e., coinfection), better discrimination of certain bacterial groups (e.g., *Enterobacteriaceae* and staphylococci), detection of antibiotic resistance and virulence determinants, and the possibility of revealing viral etiologies of infection. It is also expected to increase the sensitivity, although a recent meta-analysis of metagenomic studies reported values of sensitivity for certain types of specimens (such as cerebrospinal or synovial fluid) that were comparable to the herein-presented sensitivity of ST (for further details, see reference 9). A number of issues need to be addressed before metagenomics replaces ST in routine molecular diagnostics. Among others, these include the current long turnaround time of NGS examination and analysis, the costly run of a single sample, the requirement of specific skills in bioinformatics, and difficulties in distinguishing true pathogens from bystanders and contaminants (44).

## LIMITATIONS

Among a variety of diseases, the overall diagnostic sensitivity (79%; 95% CI, 73 to 84%) and specificity (83%; 95% CI, 72 to 90%) against those of culture and Duke standard (IE) were limited and accompanied by considerable heterogeneity (Fig. 3 to 5). A QUADAS-2 assessment of study quality showed weaknesses of all but 2 studies (28, 32) in all domains of risk-of-bias evaluation (Fig. 2). Considerable bias and concerns were noted with respect to doubts of independent interpretation of ST and reference results. A variety of systems and protocols for culture were used as standards or not described in detail. This and using another standard with IE, the modified Duke criteria, although one of its major criteria is based on microbiological testing by blood culture for organisms typical of IE, may have added to weaknesses of the present meta-analysis within and across the disease subgroups. Moreover, a lack of details of the ST procedure and the measure for prevention of contamination and reporting of false results in several studies was a point of concern. All of these factors and, finally, the selection of a variety of diseases and specimen types may have had an impact on the estimated overall accuracy and heterogeneity. A case-control design was avoided in all but one study (36). Upon exclusion of this study from the analysis, however, the results for overall accuracy were not influenced.

An inherent limitation of ST is the lack of antibiotic resistance tests. Nonetheless, the identity of pathogens provides some choice of targeted antibiotic treatment in cases where culture lacks any information. Furthermore, ST has a limited ability to discriminate mixed infections. In such cases, online tools are available that resolve mixed sequences to single-species identification (15). Another notable shortcoming of 16S rRNA gene Sanger sequencing is the limited discrimination of certain species, in particular nonhemolytic streptococci, some *Enterobacteriaceae*, and coagulase-negative staphylococci, which may have an impact on antibiotic therapy (23, 56). A further issue of the agnostic approach by ST is that it is prone to false-positive results by nonrelevant skin-colonizing and environmental species. The discrimination of pathogens from contaminants may be difficult and requires careful clinical examination—as is the practice in culture diagnosis. Clearly defined criteria, including the patient’s medical history, former and current microbiological results from other specimens, and other clinical information should always lead to deciding whether an ST-positive, culture-negative finding is of added value or not, since this is critical in the context of clinical decision-making (25, 57). Another shortcoming of ST is the long turnaround time and elaborate handling by specialized personnel. Full automation of the whole protocol would aid in further suitability of ST in clinical diagnosis.

In summary, the strengths and limitations characteristic of ST preferably direct its clinical applicability to the rapid diagnosis of culture-negative cases where infection is strongly suspected (30, 32). The benefit of ST, however, must be consolidated in terms of clinical and economic value by additional, preferably prospective, blinded studies with well predefined protocols.
